# Supplementary material for: Sand Play for 0–8-Year-Old Children’s Health and Development: A Systematic Review Protocol
Source: Int J Environ Res Public Health. 2021 Sep 26;18(19):10112. doi: 10.3390/ijerph181910112 (PMC8508018; doi:10.3390/ijerph181910112)
Supplement: Supplementary file 1 [file ijerph-18-10112-s001.zip › ijerph-1379270 Supplementary corrected file 1.pdf]

International Journal of
Environmental Research and Public Health

MDPI

Supplementary Material File 1: PRISMA-P Checklist

PRISMA-P (preferred reporting items for systematic review and meta-analysis protocols) 2015 checklist adapted for use with protocol submissions in Moher D. et al. Preferred reporting items for systematic review and meta-analysis protocols (PRISMA-P) 2015 statement. *Sys Rev,* **2015**, *4*, 1 [26]

Section/topic	#	Checklist item	Information reported		Line number(s)
			Yes	No	
**ADMINISTRATIVE INFORMATION**					
**Title**					
Identification	1a	Identify the report as a protocol of a systematic review	x		1–3
Update	1b	If the protocol is for an update of a previous systematic review, identify as such		x	
**Registration**	2	If registered, provide the name of the registry (e.g., PROSPERO) and registration number in the Abstract	x		16
**Authors**					
Contact	3a	Provide name, institutional affiliation and e-mail address of all protocol authors; provide physical mailing address of corresponding author	x		5–10
Contributions	3b	Describe contributions of protocol authors and identify the guarantor of the review	x		469–473
**Amendments**	4	If the protocol represents an amendment of a previously completed or published protocol, identify as such and list changes; otherwise, state plan for documenting important protocol amendments			N/A
**Support**					
Sources	5a	Indicate sources of financial or other support for the review			N/A
Sponsor	5b	Provide name for the review funder and/or sponsor			N/A

Role of sponsor/funder	5c	Describe roles of funder(s), sponsor(s) and/or institution(s), if any, in developing the protocol			N/A
**INTRODUCTION**					
**Rationale**	6	Describe the rationale for the review in the context of what is already known	x		29–132
**Objectives**	7	Provide an explicit statement of the question(s) the review will address with reference to participants, interventions, comparators and outcomes (PICO)	x		133–135
**METHODS**					
**Eligibility criteria**	8	Specify the study characteristics (e.g., PICO, study design, setting, time frame) and report characteristics (e.g., years considered, language, publication status) to be used as criteria for eligibility for the review	x		144–191; Supplementary material file 2.
**Information sources**	9	Describe all intended information sources (e.g., electronic databases, contact with study authors, trial registers or other grey literature sources) with planned dates of coverage	x		192–223
**Search strategy**	10	Present draft of search strategy to be used for at least one electronic database, including planned limits, such that it could be repeated	x		Supplementary Material file 3.
STUDY RECORDS					
Data management	11a	Describe the mechanism(s) that will be used to manage records and data throughout the review	x		225–230
Selection process	11b	State the process that will be used for selecting studies (e.g., two independent reviewers) through each phase of the review (i.e., screening, eligibility and inclusion in meta-analysis)	x		231–239
Data collection process	11c	Describe planned method of extracting data from reports (e.g., piloting forms, done independently, in duplicate), any processes for obtaining and confirming data from investigators	x		240–254
**Data items**	12	List and define all variables for which data will be sought (e.g., PICO items, funding sources), any pre-planned data assumptions and simplifications	x		254–273; Supplementary Material file 4

**Outcomes and prioritisation**	13	List and define all outcomes for which data will be sought, including prioritisation of main and additional outcomes, with rationale	x		268–273; Supplementary Material file 4
**Risk of bias in individual studies**	14	Describe anticipated methods for assessing risk of bias of individual studies, including whether this will be done at the outcome or study level, or both; state how this information will be used in data synthesis	x		274–312; Supplementary Material file 5
DATA					
**Synthesis**	15a	Describe criteria under which study data will be quantitatively synthesised	x		340–342
	15b	If data are appropriate for quantitative synthesis, describe planned summary measures, methods of handling data and methods of combining data from studies, including any planned exploration of consistency (e.g., I^2, Kendall's tau)	x		343–364
	15c	Describe any proposed additional analyses (e.g., sensitivity or subgroup analyses, meta-regression)			N/A
	15d	If quantitative synthesis is not appropriate, describe the type of summary planned	x		313–340; 365–400
**Meta-bias(es)**	16	Specify any planned assessment of meta-bias(es) (e.g., publication bias across studies, selective reporting within studies)			N/A
**Confidence in cumulative evidence**	17	Describe how the strength of the body of evidence will be assessed (e.g., GRADE)	x		401–416

International Journal of
Environmental Research
and Public Health

MDPI

Supplementary Material file 2. Inclusion and exclusion criteria

Screening process: Titles and abstracts will be reviewed independently by one reviewer, reasons given for exclusion and 15% checked by two reviewers.
Full text screening will require each article being independently reviewed by two reviewers. Two options are available— '**include**' or '**exclude**'—and the decision as to whether the study is eligible will require both reviewers agreeing. In full text screening it is important that reasons are given for excluding articles. Both reviewers should indicate the same primary reason for exclusion using one of the options below.

Reasons for exclusion

A reason needs to be given for each full text article that has been excluded. The primary reason for exclusion should be selected from the list below, which is a condensed list of inclusion and exclusion criteria. If there are many reasons for exclusion, select the one that you consider as primary.

- **Wrong study design:** not a primary study design
- **Wrong participants:** children > 8 years, primary/secondary school children
- **Wrong intervention/exposure:** if the study is not in sand play environment
- **Wrong outcomes**: not child-level, use of unvalidated measurement method, ignoring research ethics
- **Wrong language:** not English
- **No full text**: full text is not available after Internet search and/or requesting the first author via email

The table below provides the inclusion and exclusion criteria for the studies being screened.

	**Inclusion**	**Exclusion**
**Study designs**	All quantitative and qualitative primary research designs will be considered if outcomes were child-level and they were assessed in the sand play environment. Studies that explore perceptions from parent, educator, practitioner or child on child-level outcome when the child was in sand play environment will be included.	Study designs where the outcome measurement cannot readily be associated with the exposure and outcome, for example, those utilising sand play environments but not reporting child-level outcomes, will be excluded.
**Participants**	Based on the definition of the World Health Organization (2021) of the age at which early childhood development occurs, children aged 0 to 8 years of age will be included. Early childhood education in many countries coincides with these ages, although varies by country (e.g., Finland 0–6 years, Sweden 1–5 years; United States children from birth through state-designated compulsory school starting age of 5–8 years). In retrospective designs, parts focusing on the time the children were ≤ 8 will be included. Regarding longitudinal designs in which children have been followed at different ages, the part of the study in which the children have been aged 0–8 years will be included. Likewise, regarding research designs with groups of children of different ages or individual children of different ages, the part of the study in which the children were aged 0–8 years will be included. Reported mean age, range or median will be used to decide whether the study is eligible. If a study is conducted in an ECE setting, but no age is reported, it will be considered. Children with disease or disability conditions, among whom the association between the	Studies that include children > 8 years will be excluded. Studies that do not report that participants' mean age, range or median is ≤ 8, but report that participants are primary/secondary school children, will be excluded.

	condition and sandy environment (e.g., rehabilitation) is investigated, will be included. If a research design includes many children with disease or disability conditions, those children among whom the association between the condition and sandy environment is investigated will be included.	
**Interventions/ exposures**	Sand play is an umbrella term that encompasses different sand environments and materials. They can be traditional low-edged sandboxes of different sizes or delimited sandy areas of different sizes where children spend time, act, play, etc. Sand environments and materials built indoors will be considered. With the concept of 'play' we mean being, acting, playing, behaving, etc.—all kinds of activity or behaviours that happen in the sand environment or with sand materials. The concept of play is used because according to Pellegrini, Dupuis and Smith [12], it is a central component of a young child's life. Sand play can be included in an intervention program or in early childhood education. Sand play can also happen at home, in a neighbourhood environment, in a park, in an organisational activity aimed at children or as part of rehabilitation.	Interventions/exposures where sand environments or materials are not the exposure, i.e., the authors do not describe their studies as using sand environments or materials will be excluded.
**Comparators**	This criterion only applies for multi-group studies. Qualitative, pre/post study designs that do not have a comparison will still be included.	-
**Outcomes**	Any child-level outcome. It can relate to all aspects of health and development [29, 30]. These can include child's physical domain (e.g., physical activity, indicators of weight status, fitness, fundamental motor skills, perceptual-motor skills, fine motor skills, infectious conditions and other minor injuries), cognitive domain (e.g., attention, memory, pattern recognition, executive functions, mathematical skills) or social emotional domain (e.g., interactions, pro-social behaviour, resilience, stress) outcomes of health and development.	Studies will be excluded if they include outcomes that are not child-level (for example, impact on parents' perceptions of their own mental wellbeing) and if they do not report information on the validity and reliability of measurement methods used for investigation of the associations between sand play and child-level outcome (for both quantitative and qualitative designs). Studies that do not report on adherence to research ethics to obtain child-level outcomes will be excluded.
**Time frames for follow-up**	This applies only for multi-group studies. There will be no restrictions by time frame for follow-up.	-
**Settings (in which the interventions are delivered)**	There will be no restrictions by type of setting provided that the above criteria are met.	Settings in which outcome measurement cannot readily be associated with the exposure of interest will be excluded.
**Languages of publication**	We will include articles reported in English.	Languages other than English will be excluded
**Publication status (e.g., unpublished material or abstracts)**	Only published material will be included. Congress proceedings, book chapters, as well as grey literature such as dissertations, will be included if they report accurate data on study designs, participants, interventions/exposures and outcomes and if they include enough information on validated methods and research ethics.	Unpublished material and abstracts will be excluded. Grey literature using unvalidated measures should be excluded.<br><br>Reviewers should ensure questionnaires and other measurement methods are validated by checking the reference or conducting a quick Internet search.

**Years of publication**	There is no restriction of years of publication	-

Supplementary Material File 3: A draft ERIC search strategy

Search strategy for ERIC (Proquest) database:

S1 sand OR ‘sand pit*’ OR sandpit* OR ‘sand box*’ OR sandbox* OR ‘sand play*’ OR sand play*

S2 child* OR toddler* OR infant*

S3 AU (sand*)

S4 S1 AND S2

S5 S4 NOT S3

Supplementary Material file 4. Data extraction templates

Table. Data extraction template for quantitative studies

Study details	Participants			Intervention/exposure		Data collection methods		Findings	
Author, year, country, study design	Age (range and/or mean ± SD), separated by intervention/exposure (E) and comparator (C)	Sample size (n), gender (n or % of girls and boys) separated by intervention/exposure (E) and comparator (C)	Background information	Description	Duration, follow-up	Assessment and analaysis methods	Time point of assessment	Outcome findings (outcome unit, effect estimates, standard deviations, confidence intervals, direction of effect, statistical significance, etc.) separated by intervention/exposure and comparator	Conclusions drawn from findings by the authors of the study

Table. Data extraction template for qualitative studies

Study details		Participants			Intervention/exposure		Data collection and analysis methods		Findings	
Author, year, country, study design (e.g., ethnographic, narrative research, historical, case study, phenomenology)	Aims	Age (range and/or mean ± SD)	Sample size (n), gender (n or % of girls and boys)	Background information	Description	Duration, follow-up	Methodology	Method of analysis	Summary of findings	Conclusions drawn from findings by the authors of the study

Supplementary Material file 5. A draft of the quality assessment tools with modifications

Modifications are highlighted with red

Quality assessment tool for quantitative interventional studies

Effective Public Health Practice Project (EPHPP) Quality Assessment Tool [34]

Quality assessment Tool Quantitative Studies Dictionary. Available online: https://www.ephpp.ca/PDF/QADictionary_dec2009.pdf [45] (accessed 18th June 2021)

A) SELECTION BIAS

(Q1) Are the individuals selected to participate in the study likely to be representative of the target population? (i.e., children aged 0–8 years)

1. Very likely
2. Somewhat likely
3. Not likely
4. Can't tell

(Q2) What percentage of selected individuals (consented by parent(s))

1. 80–100% agreement
2. 60–79% agreement
3. Less than 60% agreement
4. Not applicable
5. Can't tell

RATE THIS SECTION	STRONG	MODERATE	WEAK
See dictionary	1	2	3

B) STUDY DESIGN

Indicate the study design:

1. Randomised controlled trial
2. Controlled clinical trial
3. Cohort analytic (two group pre + post)
4. Case-control
5. Cohort (one group pre + post (before and after))
6. Interrupted time series
7. Other (specify) ____________________________
8. Can't tell

Was the study described as randomised? If No, go to Component C.

No Yes

If Yes, was the method of randomisation described? (See dictionary)

No Yes

If Yes, was the method appropriate? (See dictionary)

No Yes

RATE THIS SECTION	STRONG	MODERATE	WEAK
See dictionary	1	2	3

C) CONFOUNDERS

(Q1) Were there important differences between groups prior to the intervention?

1. Yes
2. No
3. Can't tell

The following are examples of confounders (confounders in this review highlighted with red):

1. Race
2. Sex
3. Marital status/family
4. Age
5. SES (income or class)
6. Education
7. Health status
8. Pre-intervention score on outcome measure

(Q2) If yes, indicate the percentage of relevant confounders that were controlled (either in the design (e.g., stratification, matching) or analysis):

1. 80–100% (all confounders)
2. 60–79% (two confounders)
3. Less than 60% (one confounder)
4. Can't tell

RATE THIS SECTION	STRONG	MODERATE	WEAK
See dictionary	1	2	3

D) BLINDING

(Q1) Was (were) the outcome assessor(s) aware of the intervention or exposure status of participants?

1. Yes
2. No
3. Can't tell

(Q2) Were the study participants aware of the research question?

1. Yes
2. No
3. Can't tell

RATE THIS SECTION	STRONG	MODERATE	WEAK
See dictionary	1	2	3

E) DATA COLLECTION METHODS

(Q1) Were data collection tools shown to be valid?

1. Yes
2. No
3. Can't tell

(Q2) Were data collection tools shown to be reliable?

1. Yes

2. No
3. Can’t tell

RATE THIS SECTION	STRONG	MODERATE	WEAK
See dictionary	1	2	3

F) WITHDRAWALS AND DROP-OUTS

(Q1) Were withdrawals and drop-outs reported in terms of numbers and/or reasons per group?

1. Yes
2. No
3. Can’t tell
4. Not applicable (i.e., one-time surveys or interviews)

(Q2) Indicate the percentage of participants completing the study (if the percentage differs by groups, record the lowest).

1. 80–100%
2. 60–79%
3. Less than 60%
4. Can’t tell
5. Not applicable (i.e., retrospective case-control)

RATE THIS SECTION	STRONG	MODERATE	WEAK
See dictionary	1	2	3

G) INTERVENTION INTEGRITY (this component will not be included in the global rating of the EPHPP)

(Q1) What percentage of participants received the allocated intervention or exposure of interest?

1. 80–100%
2. 60–79%
3. Less than 60%
4. Can’t tell

(Q2) Was the consistency of the intervention measured?

1. Yes
2. No
3. Can’t tell

(Q3) Is it likely that subjects received an unintended intervention (contamination or co-intervention) that may influence the results?

1. Yes
2. No
3. Can’t tell

H) ANALYSES (this component will not be included in the global rating of the EPHPP)

(Q1) Indicate the unit of allocation (circle one) (intervention/exposure allocation)

- community (e.g., public playground, neighbourhood area)
- organisation/institution (e.g., ECE unit)
- practice/office (i.e., with a therapist/rehabilitation unit)
- individual (individual child)

(Q2) Indicate the unit of analysis (circle one)

- community (e.g., public playground, neighbourhood area)
- organisation/institution (e.g., ECE unit)
- practice/office (i.e., with a therapist/rehabilitation unit)
- individual (individual child)

(Q3) Are the statistical methods appropriate for the study design?

1. Yes
2. No
3. Can't tell

(Q4) Is the analysis performed by intervention allocation status (i.e., intention to treat) rather than the actual intervention received?

1. Yes
2. No
3. Can't tell

COMPONENT RATINGS

Please transcribe the information from the grey boxes on pages 1–3 onto this page. See dictionary on how to rate this section.

A	SELECTION BIAS	STRONG	MODERATE	WEAK
		1	2	3
B	STUDY DESIGN	STRONG	MODERATE	WEAK
		1	2	3
C	CONFOUNDERS	STRONG	MODERATE	WEAK
		1	2	3
D	BLINDING	STRONG	MODERATE	WEAK
		1	2	3
E	DATA COLLECTION METHOD	STRONG	MODERATE	WEAK
		1	2	3
F	WITHDRAWALS AND DROPOUTS	STRONG	MODERATE	WEAK
		1	2	3

GLOBAL RATING FOR THIS PAPER (circle one):

1 STRONG (no WEAK ratings)
2 MODERATE (one WEAK rating)
3 WEAK (two or more WEAK ratings)

With both reviewers discussing the ratings:

Is there a discrepancy between the two reviewers with respect to the component (A–F) ratings?

No Yes

If yes, indicate the reason for the discrepancy

1 Oversight
2 Differences in interpretation of criteria
3 Differences in interpretation of study

Final decision of both reviewers (circle one):

1 STRONG
2 MODERATE
3 WEAK

In addition to the EPHPP component ratings as described above, component ratings of this paper will be converted to into percentages as follows:

A	SELECTION BIAS	STRONG	MODERATE	WEAK
		1 -> 3	2 -> 2	3 -> 1
B	STUDY DESIGN	STRONG	MODERATE	WEAK
		1 -> 3	2 -> 2	3 -> 1
C	CONFOUNDERS	STRONG	MODERATE	WEAK
		1 -> 3	2 -> 2	3 -> 1
D	BLINDING	STRONG	MODERATE	WEAK
		1 -> 3	2 -> 2	3 -> 1
E	DATA COLLECTION METHOD	STRONG	MODERATE	WEAK
		1 -> 3	2 -> 2	3 -> 1
F	WITHDRAWALS AND DROPOUTS	STRONG	MODERATE	WEAK
		1 -> 3	2 -> 2	3 -> 1
Potential total score range is 6–18			Total score =	
Convert to a percentage as follows:<br>total score/18 × 100 = %			Total score as percentage =	

Quality assessment tool for cross-sectional studies

The National Heart, Lung and Blood Institute (NHLBI) Quality Assessment Tool for Observational Cohort and Cross-Sectional Studies [35]

Criterion	Yes	No	Other (~~CD~~, NR, NA)*
1. Was the research question or objective in this paper clearly stated?			
2. Was the study population clearly specified and defined?			
3. Was the participation rate of eligible persons at least 50%?			
4. Were all the subjects selected or recruited from the same or similar populations (including the same time period)? Were inclusion and exclusion criteria for being in the study prespecified and applied uniformly to all participants?			
5. Was a sample size justification, a power description or variance and effect estimates provided?			
6. For the analyses in this paper, were the exposure(s) of interest measured prior to the outcome(s) being measured? (e.g., a participant-child was observed in the sand environment at baseline)			
7. Was the timeframe sufficient so that one could reasonably expect to see an association between exposure and outcome if it existed?			
8. For exposures that can vary in amount or level, did the study examine different levels of the exposure as related to the outcome (e.g., categories of exposure or exposure measured as continuous variable)?			
9. Were the exposure measures (independent variables) clearly defined, valid, reliable and implemented consistently across all study participants? (i.e., appropriate validation of measurement methods used)			
10. Was the exposure(s) assessed more than once over time?			
11. Were the outcome measures (dependent variables) clearly defined, valid, reliable and implemented consistently across all study participants?			
12. Were the outcome assessors blinded to the exposure status of participants?			
13. Was loss to follow-up after baseline 20% or less?			
14. Were key potential confounding variables measured and adjusted statistically for their impact on the relationship between exposure(s) and outcome(s)? (key potential confounders: age, gender, health status)			

*~~CD, cannot determine;~~ NA = not applicable; NR = not reported; If there is insufficient detail reported in a paper or the reviewers cannot not determine if the answer is ‘yes’ or ‘no’ for a criterion, the original study authors will be contacted via email for more information. If information is not received from the authors of the paper within a month, the answer ‘no’ will be assumed for that criterion.

In addition to the NHLBI overall quality rating for this paper as described above, it will be converted to into percentages as described below. If a criterion is not applicable to this paper, it will be disregarded in the total scoring.

Criterion	Yes	No	NR → Assume 'No' if NR information is not received from the study authors	NA
1. Was the research question or objective in this paper clearly stated?				
2. Was the study population clearly specified and defined?				
3. Was the participation rate of eligible persons at least 50%?				
4. Were all the subjects selected or recruited from the same or similar populations (including the same time period)? Were inclusion and exclusion criteria for being in the study prespecified and applied uniformly to all participants?				
5. Was a sample size justification, a power description or variance and effect estimates provided?				
6. For the analyses in this paper, was the exposure(s) of interest measured prior to the outcome(s) being measured (e.g., a participant-child was observed in the sand environment at baseline)?				
7. Was the timeframe sufficient so that one could reasonably expect to see an association between exposure and outcome if it existed?				
8. For exposures that can vary in amount or level, did the study examine different levels of the exposure as related to the outcome (e.g., categories of exposure or exposure measured as a continuous variable)?				
9. Were the exposure measures (independent variables) clearly defined, valid, reliable and implemented consistently across all study participants? (i.e., appropriate validation of measurement methods used)				
10. Was the exposure(s) assessed more than once over time?				
11. Were the outcome measures (dependent variables) clearly defined, valid, reliable and implemented consistently across all study participants?				
12. Were the outcome assessors blinded to the exposure status of participants?				
13. Was loss to follow-up after baseline 20% or less?				
14. Were key potential confounding variables measured and adjusted statistically for their impact on the relationship between exposure(s) and outcome(s) (key potential confounders: age, gender, health status)?				

Total scoring:<br>The potential maximum score of this paper is equal to the total number of applicable criteria (/14)	Total score =
Convert to a percentage as follows: number of 'Yes' answers given by a reviewer/potential maximum number of 'Yes' answers × 100 = %	Total score as percentage =

Note: NA = not applicable; NR = not reported

International Journal of Environmental Research and Public Health

MDPI

Quality assessment tool for qualitative studies

Standards for Reporting Qualitative Research (SRQR). Available online: http://www.equator-network.org/reporting-guidelines/srqr/ [36] (accessed 18th June 2021).

Supplemental digital content for O'Brien, B.; Harris, I.B.; Beckman, T.J.; Reed, D.A.; Cook, D.A. Standards for Reporting Qualitative Research. *Academic Medicine*, 2014, *89*, 1245–1251. [36]

The quality of a paper will be assessed with this tool numerically by giving one point for each entry in the right-hand column of the table. Thus, the range of scores describing the quality of a paper may be 0–21. The score 21 means that a paper supports all 21 items. A study will be excluded if the paper does not support items 1 to 19. The justification for the exclusion of a study based on these limitations is because the results would not be reliable, for example if the conducting of the study was not described with sufficient accuracy to ensure the validity and reliability of the methods used.

Title and abstract	Page/line no.(s)
1. Title: Concise description of the nature and topic of the study identifying the study as qualitative or indicating the approach (e.g., ethnography, grounded theory) or data collection methods (e.g., interview, focus group) is recommended	
2. Abstract: Summary of key elements of the study using the abstract format of the intended publication; typically includes background, purpose, methods, results and conclusions	
Introduction	
3. Problem formulation: Description and significance of the problem/phenomenon studied; review of relevant theory and empirical work; problem statement	
4. Purpose or research question: Purpose of the study and specific objectives or questions	
Method	
5. Qualitative approach and research paradigm: Qualitative approach (e.g., ethnography, grounded theory, case study, phenomenology, narrative research) and guiding theory if appropriate; identifying the research paradigm (e.g., postpositivist, constructivist/interpretivist) is also recommended (guiding theory not necessary)	
6. Researcher characteristics and reflexivity: Researchers' characteristics that may influence the research, including personal attributes, qualifications/experience, relationship with participants, assumptions and/or presuppositions; potential or actual interaction between researchers' characteristics and the research questions, approach, methods, results and/or transferability	
7. Context: Setting/site and salient contextual factors	
8. Sampling strategy: How and why research participants, documents or events were selected; criteria for deciding when no further sampling was necessary (e.g., sampling saturation)	
9. Ethical issues pertaining to human subjects: Documentation of approval by an appropriate ethics review board and participant consent, or explanation for lack thereof; other confidentiality and data security issues	
10. Data collection methods: Types of data collected; details of data collection procedures including (as appropriate) start and stop dates of data collection and analysis, iterative process, triangulation of sources/methods and modification of procedures in response to evolving study findings	
11. Data collection instruments and technologies: Description of instruments (e.g., interview guides, questionnaires) and devices (e.g., audio recorders) used for data collection; if/how the instrument(s) changed over the course of the study	
12. Units of study: Number and relevant characteristics of participants, documents or events included in the study; level of participation (could be reported in results)	

13. Data processing: Methods for processing data prior to and during analysis including transcription, data entry, data management and security, verification of data integrity, data coding and anonymisation/de-identification of excerpts	
14. Data analysis: Process by which inferences, themes, etc., were identified and developed, including the researchers involved in data analysis; usually references a specific paradigm or approach	
15. Techniques to enhance trustworthiness: Techniques to enhance trustworthiness and credibility of data analysis (e.g., member checking, audit trail, triangulation)	
Results/Findings	
16. Synthesis and interpretation: Main findings (e.g., interpretations, inferences and themes); might include development of a theory or model, or integration with prior research or theory	
17. Links to empirical data: Evidence (e.g., quotes, field notes, text excerpts, photographs) to substantiate analytic findings	
Discussion	
18. Integration with prior work, implications, transferability and contribution(s) to the field: Short summary of main findings; explanation of how findings and conclusions connect to, support, elaborate on or challenge conclusions of earlier scholarship; discussion of scope of application/generalisability; identification of unique contribution(s) to scholarship in a discipline or field	
19. Limitations: Trustworthiness and limitations of findings	
20. Conflicts of interest: Potential sources of influence or perceived influence on study conduct and conclusions; how these were managed (this item may not be applicable for a study)	
21. Funding: Sources of funding and other support; role of funders in data collection, interpretation and reporting	
Quality appraisal of a qualitative study: 21 marks in the right column	
Total quality score =	
